# Noninvasive Prediction of TERT Promoter Mutations in High-Grade Glioma by Radiomics Analysis Based on Multiparameter MRI

**DOI:** 10.1155/2020/3872314

**Published:** 2020-05-15

**Authors:** Hongan Tian, Hui Wu, Guangyao Wu, Guobin Xu

**Affiliations:** Department of Radiology, Zhongnan Hospital of Wuhan University, Wuhan 430071, China

## Abstract

**Objectives:**

To investigate the predictors of telomerase reverse transcriptase (TERT) promoter mutations in adults suffered from high-grade glioma (HGG) through radiomics analysis, develop a noninvasive approach to evaluate TERT promoter mutations.

**Methods:**

126 adult patients with HGG (88 in the training cohort and 38 in the validation cohort) were retrospectively enrolled. Totally 5064 radiomics features were, respectively, extracted from three VOIs (necrosis, enhanced, and edema) in MRI. Firstly, an optimal radiomics signature (Radscore) was established based on LASSO regression. Secondly, univariate and multivariate logistic regression analyses were performed to investigate important potential variables as predictors of TERT promoter mutations. Besides, multiparameter models were established and evaluated. Eventually, an optimal model was visualized as radiomics nomogram for clinical evaluations.

**Results:**

6 radiomics features were selected to build Radscore signature through LASSO regression. Among them, 5 were from necrotic VOIs and 1 was from enhanced ones. With univariate and multivariate analysis, necrotic volume percentages of core (CNV), Age, Cho/Cr, Lac, and Radscore were significantly higher in TERTm than in TERTw (*p* < 0.05). 4 models were built in our study. Compared with Model B (Age, Cho/Cr, Lac, and Radscore), Model A (Age, Cho/Cr, Lac, Radscore, and CNV) has a larger AUC in both training (0.955 vs. 0.917, *p* = 0.049) and validation (0.889 vs. 0.868, *p* = 0.039) cohorts. It also has higher performances in net reclassification improvement (NRI), integrated discrimination improvement (IDI), and decision curve analysis (DCA) evaluation. Conclusively, Model A was visualized as a radiomics nomogram. Calibration curve shows a good agreement between estimated and actual probabilities.

**Conclusions:**

Age, Cho/Cr, Lac, CNV, and Radscore are important indicators for TERT promoter mutation predictions in HGG. Tumor necrosis seems to be closely related to TERT promoter mutations. Radiomics nomogram based on multiparameter MRI and CNV has higher prediction accuracies.

## 1. Introduction

As the most common primary brain tumor in adults, glioma can be classified as low-grade (LGG, WHO I-II) and high-grade (HGG, WHO III-IV) according to the classification criteria of the World Health Organization (WHO) [1]. Compared with LGG, HGG is characterized by more vigorous cell growth, more tumor angiogenesis, higher heterogeneity, and worse prognosis, especially glioblastoma (GBM), with a median survival of 12-14 months for the patients receiving standard treatments [2, 3]. As indicated in previous studies, hyperproliferativeness provides a significant biological basis for the occurrence, migration, diffusion, invasion, postoperative recurrence, and drug resistance of HGG, where the stability and nonshrinkage of telomeres play a crucial role [4].

Telomeres, controlling the limited division of normal cells, are shortened with each division of normal cells, whereas they could be continuously elongated by telomerase in cancer cells [5]. Telomerase consisted of the RNA subunit and reverse transcriptase (TERT), maintaining the length of telomere by adding hexamer repeats at the end of chromosomes [6]. Therefore, TERT plays a crucial role in the cancerization. Recently, the recurrent mutations at two hotspots termed as C228T and C250T in the TERT promoter have been identified in gliomas [7]. Besides, the mutations have been considered as one of the major mechanisms of telomerase activation in gliomas [8]. Up to now, a large number of researches have explored the role and value of TERT in glioma, which demonstrated that 80% of the TERT promoters in the primary GBM have mutated [9]. The TERT genotype is not only a vital prognostic and predictive biomarker for glioma, especially for HGG [10, 11], but also a promising indicator for the sensitivity of GBM to radiotherapy and temozolomide [12]. Currently, the TERT genotype has been prevailingly determined by sequencing tumor samples, which can only be obtained postoperatively. Accurate assessment of the TERT genotype before surgery can direct the exploitation of therapeutic strategies. Therefore, the establishment of noninvasive technology to identify the TERT genotype of tumors is urgently needed.

Radiomics has attracted increased attention in recent years due to the representation of medical images containing information on the pathophysiology and prognosis of diseases [13]. A variety of quantitative radiological features could be extracted from medical images to reveal the information on tumors [14]. Radiomics feature has been applied as the noninvasive alternative to identify the genomic and proteomic changes in tumors, which also broadly utilized in tumor diagnosis, prognosis prediction, treatment selection, gene prediction, and so on [15–18]. However, the use of radiomics analysis to predict the mutant status of the TERT promoter has not been widely reported. This study aimed to investigate the predictors of TERT promoter mutations in HGG through radiomics analysis and develop a noninvasive approach to evaluate of TERT promoter mutations.

## 2. Materials and Methods

The institutional review board has approved this retrospective study, and the requirements for patient informed consent were waived for the anonymity of data.

### 2.1. Patients

**Figure 1:**
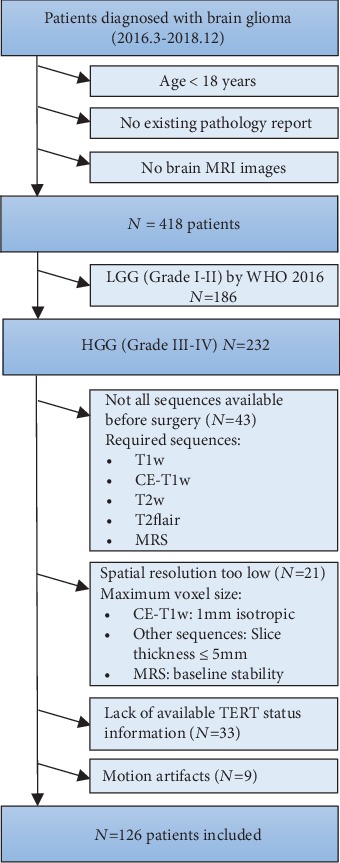
Flowchart of patient inclusion. LGG: low-grade glioma; HGG: high-grade glioma; T1w: T1 weighted; CE: contrast-enhanced; Flair: fluid-attenuated inversion recovery; MRS: magnetic resonance spectroscopy.

Based on the inclusion and exclusion criteria as shown in Figure 1, 126 patients were finally enrolled in our study. All the subjects were randomly divided into the training and the validation cohorts by computer sampling at a ratio of 7 : 3. The TERT promoter mutations were determined by capillary electrophoresis. Clinical and pathological data were obtained via reviewing electronic medical records.

### 2.2. Data Acquisition of MRI

All preoperative MRI was performed on 3.0 T MR scanners (Magneto Trio, Siemens, Germany) with an eight-channel head coil. Contrast-enhanced T1-weighted (CE-T1w), T2-weighted imaging fluid-attenuated inversion recovery (T2flair), T1-weighted (T1w), T2-weighted (T2w), and magnetic resonance spectroscopy (MRS) sequences were applied for the following analyses. The acquisition parameters were summarized in Supplementary S1.

### 2.3. Preprocessing, Segmentation, and Feature Extraction of Images

The schemes of image preprocessing consisted of coregistration, reslice, and normalization. Firstly, the T2flair, T2w, and T1w images were coregistered to the corresponding CE-T1w images on the basis of affine transformation through the Linear Image Registration Tool (FLIRT) of Functional MRI of the Brain (FMRIB) Software Library (FSL) of the Oxford Center. Subsequently, the resolution of each modality was uniformly resampled to 1 mm × 1mm × 1mm. Finally, the intensity of the images of each modality was normalized according to the Collewet normalization algorithm (mean ± 3 sigmas) to correct the effects of different acquisition protocols [19]. The success of coregistration and normalization was visually verified by two authors (T.H.A. and W.H., with 10 and 3 years of experience in brain MRI research, respectively).

**Figure 2:**
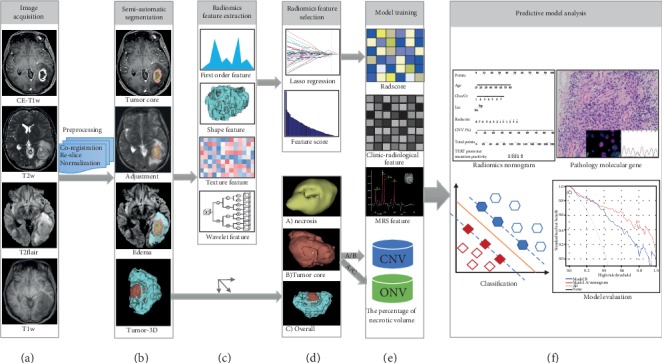
Flowchart of the radiomics analysis. (a) Image data acquisition included CE-T1w, T2flair, T2w, and T1w sequences. And then, data preprocessing: coregistration, reslice, and normalization. (b) The volume of interests (VOIs) of the tumor lesion and peritumoral edema regions were drawn by semiautomatic segmentation. (c) Radiomics features were extracted, including first-order feature, shape-based feature, texture feature, and wavelet feature. (d) Discriminative features were selected by the LASSO regression analysis. (e) The model was trained by radiomics features, clinical features, MRS features, and the percentage of necrotic volume. (f) Radiomics nomogram was established for predicting TERT promoter mutations in adults with high-grade gliomas. The ROC, calibration, and DCA curves were performed for further statistical analyses.

**Figure 3:**
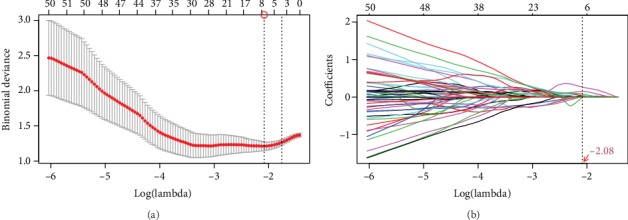
Radiomics feature selection using LASSO. (a) Selection of the optimal value of lambda (*λ*). Tuning log(*λ*) selection in the LASSO model used to perform 5-fold cross-validation via the minimum criteria. (b) The LASSO coefficient profiles included 1230 features. The vertical line was drawn at the selected log(*λ*) above, and 6 features with nonzero coefficients were finally identified.

Tumor segmentation was conducted on 3D slicer (version 4.10.1). As illustrated in Figures 2 and 3 heterogeneous regions (the enhanced lesion inclusive of necrosis (tumor core), enhanced, and necrosis) were plotted by the semiautomatic segment editor module. Detailed procedures and parameters for tumor separation were listed in Supplementary S2 to facilitate reproducibility. The volume of interest (VOI) was taken charge by T.H.A., who was trained and supervised by a board-certified radiologist specialized in neurooncology (W.G.Y. with 25-year experience). All the VOIs were eventually registered to the MNI152 standard space for obtaining the location information by FSL-FLIRT.

The radiomics features were extracted by PyRadiomics (version 2.1.2), a flexible open-source platform capable of obtaining a large panel of engineered features from medical images [20]. The extracted features included the “first-order statistics (First-order), Gray Level Cooccurrence Matrix (GLCM), Gray Level Dependence Matrix (GLDM), Gray Level Run Length Matrix (GLRLM), Gray Level Size Zone Matrix (GLSZM), Neighbouring Gray Tone Difference Matrix (NGTDM), Shap3D and Shap2D.” All the above features were displayed in the Supplementary S3 to facilitate the application of our findings. Only the features of T2flair and CE-T1w sequences were acquired, which were considered as the best series of HGG studies in various former studies [21]. Therefore, we obtained the features of necrosis, enhanced, and edema VOIs on the above two sequences, respectively.

### 2.4. Intraobserver and Interobserver Agreement

Interobserver and intraobserver agreement of VOI-based radiomics feature in 30 randomly chosen patients were evaluated in our study. Interobserver agreement of feature extraction by two authors (T.H.A. and W.H.) was initially analyzed. Meanwhile, to assess the intraobserver agreement, one of the researchers (T.H.A.) repeated the extraction twice in two weeks following the equivalent protocol. These radiomics features extracted from the VOIs were evaluated by the intraclass correlation coefficient (ICC). The score of ICC greater than 0.85 was considered a satisfactory agreement.

### 2.5. Dimensionality Reduction and Radiomics Features Selection

To reduce the dimension and decrease the redundant information, two steps were scheduled to select the features. Firstly, all features were analyzed by the independent samples *t* test or Mann-Whiney *U* test in the training cohort, and variables with *p* < 0.05 were chosen as the potentially important parameters. Additionally, the least absolute shrinkage and selection operator (LASSO) feature selection algorithm was subsequently conducted for dimensionality reduction and feature selection. Nonzero coefficients chosen by LASSO as the optimal features were utilized to establish the Radscore formula, applying to calculate the Radscore for each patient to predict the TERT promoter mutations.

### 2.6. The Selection of Clinical and Radiological Characteristics

The clinical characteristics of age, gender, and tumor grade were identified. The radiological features were assessed by T.H.A., containing tumor location, the volume of the necrotic or cystic part of the tumor (NeV), the volume of the solid portion of the tumor (EnV), the volume of edema around tumor (EdV), the percentage of necrotic volume in core volume of the tumor (CNV, %) and that in overall (ONV, %), and MRS features based on enhanced region. CNV was represented as NeV/(NeV+EnV), and ONV was represented as NeV/(NeV+EnV+EdV), whose definition can be more intuitively shown in Figures 2(d) and 2(e). Univariate and multivariate logistic regression analyses were performed to determine potential important variables. The features with *p* < 0.05 were selected as the elements for the establishment of the model.

### 2.7. Development of Radiomics Model and Evaluation of the Performance

Models were established based on the logistic regression with forwarding stepwise selection. To determine the best model, the performances of predictive models were assessed in validation cohorts. The discrimination was evaluated by the receiver operating characteristic (ROC) curve, net reclassification improvement (NRI), and integrated discrimination improvement (IDI), and the clinical availability was appraised by decision curve analysis (DCA) [22–24]. To offer an individualized and easy-to-operate tool for noninvasive prediction of the TERT genotype, the optimal model was visualized as radiomics nomogram.

### 2.8. Statistical Analysis

All statistical analyses were performed according to the *R* (version 3.4.3). Data were presented as mean ± SD for continuous variables and as frequency (%) for categorical variables. Detailed statistical steps and *R* packages were listed in Supplementary S4.

## 3. Results

### 3.1. Clinical Characteristics of the Patients

126 patients who suffered from HGG were divided into the training cohort (88, 70%) and the validation cohort (38, 30%). There was no significant difference in terms of age (*p* = 0.986), gender (*p* = 0.975), grade (*p* = 0.327), or location (*p* = 0.421) between the training and validation cohorts (Table 1).

### 3.2. Feature Extraction and Dimensionality Reduction

Eventually, 1688 features were extracted from each VOI, and a total of 5064 radiomics features were generated from every patient. Totally, 1230 features (*p* < 0.05) were screened out as the potentially crucial variables at the first step of dimensionality reduction. Applying the optimal regulation weight *λ* (log(*λ*) = −2.084698) for the LASSO algorithm, 6 nonzero coefficient features were finally chosen (Figure 3). Among them, 5 were from necrotic VOIs and 1 was from enhanced ones. Three, two, and one radiomics features were selected from CE-T1w, T2flair, and original shape class, respectively (Table 2). In the end, the Radscore formula was established according to the coefficients of the six features obtained previously (Supplementary S5).

### 3.3. Interobserver and Intraobserver Agreement

**Figure 4:**
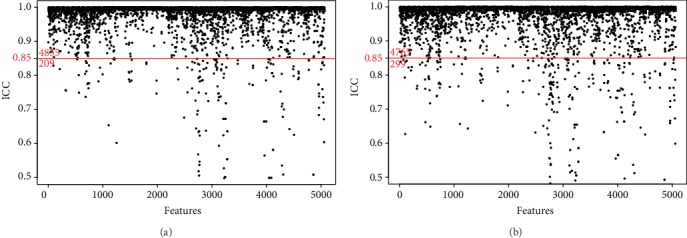
Characteristic stability evaluated by the interclass correlation coefficient (ICC). (a) 209 characteristics had poor stability in the intraobserver agreement analysis (below the red cutoff line). (b) 299 characteristics were unqualified in the interobserver agreement analysis.

For the intraobserver agreement, 209 features were unqualified (ICC <0.85), accounting for 4.13% of all variables (209/5064) (Figure 4(a)). For the interobserver agreement, 299 features were unqualified, occupying 5.91% of the variables (299/5064) (Figure 4(b)). Furthermore, none of the unqualified features consisted of the six features selected by LASSO regression (ICC were all higher than 0.85, Table 2).

### 3.4. Radiomics Model Construction and Validation

As indicated in Table 3, Radscore, Age, CNV, Cho/Cr, and Lac were significantly higher in TERTm than in TERTw (*p* < 0.05) in the training cohort by univariate and multivariate analysis.

**Figure 5:**
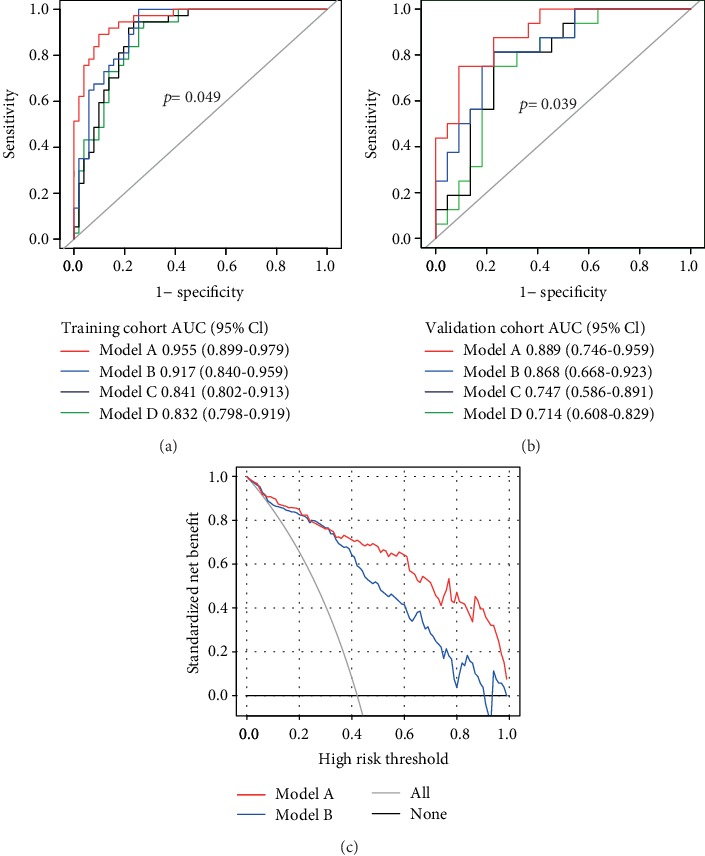
Evaluation of model performance. (a) The ROC curves of 4 models in the training cohort. (b) The ROC curves of 4 models in the validation cohort. The *p* value indicated the DeLong test for AUC of Model A and Model B. (c) The DCA curves in the validation cohort. The gray line indicated the assumption that all patients were treated. The thin black line represents the assumption that all patients had the wild of the TERT promoter.

4 models were built in this study, Age, Cho/Cr, Lac, and Radscore were taken into consideration in Model B, while Model A further added the feature of CNV based on Model B. Model C was composed of a single Radscore feature, while Model D consisted of a single CNV feature. (In fact, we had built a total of 15 models using the above 5 features (Supplementary S6)). As described in Table 4, Model A and Model B performed better, and in order to compare the differences between the two, we conducted a further evaluation. The difference of AUCs in the two models was statistically significant analyzed by the DeLong test (*p* < 0.05) (Figures 5(a) and 5(b)). Additionally, NRI was 0.187 (95% CI, 0.022-0.351) in the training cohort, 0.261 (95% CI, 0.045-0.478) in the validation cohort. Besides, the IDI was 0.146 (95% CI, 0.071-0.221) in training cohort, 0.128 (95% CI, 0.033-0.222) in the validation cohort. The DCA curves illustrated that Model A offered a higher overall net benefit in contrast to Model B, indicating Model A was superior across nearly the entire range of pt values (Figure 5(c)).

### 3.5. The Visualization of Radiomics Nomogram

**Figure 6:**
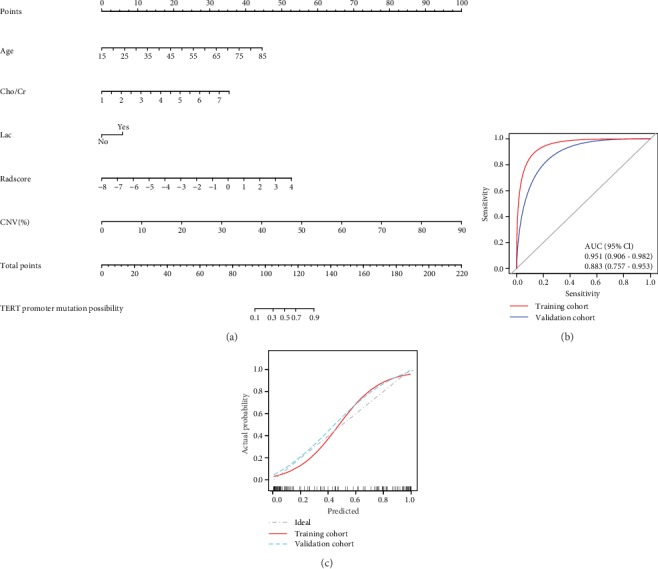
Establishment and assessment of radiomics nomogram. (a) Model A was visualized as radiomics nomogram with age, Cho/Cr, Lac, Radscore, and CNV. (b) The AUC of nomogram in training and validation cohort were both higher than 0.85, with proper discrimination. (c) The calibration curves of the nomogram in the training and validation cohort both indicated good agreement between the predictability of TERT promoter mutation and actual status. The bootstrap resampling method was adopted for the significance test of AUC and the drawing of calibration curves (times = 500). AUC: the area under the receiver operating characteristic curve.

From the above, Model A was visualized as the radiomics nomogram (Figure 6(a)). The AUC of the nomogram was 0.951 (95% CI, 0.906-0.982) in the training cohort, 0.883 (95% CI, 0.757-0.953) in the validation cohort (Figure 6(b)). Moreover, the calibration curves of the nomograms in training and validation cohorts both indicated good agreement between the predictability of TERT promoter mutations and actual status, respectively (Figure 6(c)).

## 4. Discussions

In this study, we found that the radiomics method can well predict TERT promoter mutations. Radiogenomics was a new field for studying the relationship between radiological features and genomic data, which explored the relationship between radiological features and gene phenotypes by extracting quantitative information on a large number of radiological data features [13]. Studies predicting glioma genes based on radiomics mainly focused on the IDH, 1p/19q, MGMT, ATRX, and EGFR genes, which all agreed that radiomics was an excellent noninvasive method for predicting the genetic status of glioma [14, 25, 26]. However, the relevant researches using radiomics to predict TERT genotypes were relatively rare. Arita et al. [27] used radiomics methods to predict IDH and TERT genes in grade II/III gliomas, suggesting that conventional MRI-based radiomics could be a noninvasive diagnostic technique for molecular characterization of grade II/III gliomas. As Gillies states [13], “radiomics: images are data, not just pictures,” radiomics analysis will play an increasingly important role in clinical work.

The LASSO regression was adopted to decrease redundant radiomics features. By shrinking irrelevant variables to zero and only maintaining useful features, LASSO could effectively reduce the number of variables for model fitting, which have been demonstrated to be available for high dimensional data [18, 28]. Eventually, 6 radiomics features were selected from the multiparametric and multiregional MR images in our study. With the information on regional angiogenesis and the destruction of the blood-brain barrier, the CE-T1w sequence can well display the active and necrotic areas of the tumor. Moreover, the anatomical information of the tumor can be obtained from the T2flair sequence, such as peritumoral edema [29]. In our study, 5 out of the 6 radiomics features were associated with necrosis, including 3 GLSZM features, 1 GLDM feature, and 1 shape-based feature. GLSZM features are high-order ones, which play a significant role in measuring the local heterogeneity of tumor in respect of size and grayscale. In previous studies, it has been substantiated that the information reflected by GLSZM features is most similar to the details observed by doctors at the time of manual film reading [30]. It is considered to be an important radiomics feature for the prediction of GBM survival [3]. GLDM refers to the adjacent gray correlation feature of the matrix, which reflects the gray homogeneity of the local focus. These two groups of features are common in suggesting a significant difference in the volume and grayscale of the necrotic area between the TERTm cohort and the TERTw cohort. As a statistic of grayscale texture complexity, GLCM indicates the heterogeneity and malignancy of the enhancement region. Wavelet filters are effective in improving the texture features of images significantly. An analysis led us to find out that the radiomics features of necrosis, especially GLSZM and GLDM have a potential to be closely associated with TERT promoter mutations. For further verification, nevertheless, a larger sample size and multiple centers are still required.

Yamashita et al. [31] investigated 112 GBM patients with IDH wild type based on radiomics and revealed that TERT promoter mutations were correlated with the percentage of necrotic volume and age. Our further research demonstrated that CNV dramatically upregulated in TERTm than TERTw, rather than ONV, probably because the latter contained edema volume, which was more susceptible to steroids and antihydrophobic agents [32]. To verify the discovery, 4 models were established, and there was only one more factor (CNV) in Model A than Model B. The AUC of Model A in both training cohort and validation cohort were higher than that of Model B in the ROC analyses (0.955 vs 0.917, 0.889 vs 0.868, respectively). Performing NRI and IDI analyses in both training cohort and validation cohort, the results also illustrated that Model A had a remarkable improvement over Model B (NRI >0, IDI >0). Additionally, the area under the DCA curve of Model A was larger, and the clinical net benefit was better. Therefore, it was reasonable to believe that CNV was an independent predictor for TERT promoter mutations. Various previous researches provided a rational explanation that the levels of epidermal growth factor receptor amplification and interleukin 6 could be upregulated by TERT promoter mutations, inducing tumor angiogenesis and necrosis [33].

**Figure 7:**
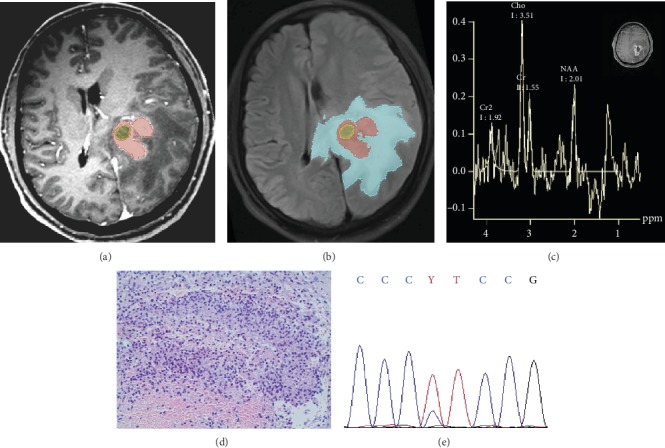
Representative images: TERT genetic status of HGG was well predicted by the radiomics nomogram. Male, 64 years old. (a) CE-T1w image. (b) T2flair image, CNV = 36%, Radscore = 2.28. (c) MRS features, Cho/cr = 2.26, Lac peak presented “M” pattern appears at 1.32 ppm. (d) Pathological hematoxylin and eosin staining image at ×20 magnification. (e) Gene sequencing revealed mutations in the TERT promoter. Applying radiomics nomogram for prediction, the probability of TERT promoter mutation was close to 0.9.

MRS is a noninvasive MR-based imaging technique that provides data on cellular metabolism. Plenty of published studies have confirmed that MRS can not only improve the diagnostic accuracy of glioma, grade the tumor, but also identify the radioactive necrosis and recurrence and predict survival rate [34]. There was a strong connection between the Cho/Cr and Lac and TERTm in our study. Cho peak, located at 3.22 ppm, was an indicator of myelination, cell metabolism, and glial hyperplasia. The increase of choline suggested an enhancement of cell membrane conversion, which was a sign of accelerated cell proliferation [35]. The concentration of creatine in brain tissue and the position of its peak in the spectral line were relatively consistent. The peak was located at 3.02 ppm, which was frequently taken as a control value. The increasing level of Cho/Cr indicated that the tumor cells were active in proliferation, which made the malignancy of the tumor predictable to some extent [36]. Lac peak, located at 1.32 ppm, was the product of anaerobic glycolysis and could not be measured in normal brain tissue. It was demonstrated in previous studies that Lac peak is more frequent to appear in HGG, which is supposedly attributed to the inhibition of aerobic respiration and cerebral tissue ischemia and hypoxia [36, 37]. According to our findings, the Lac peak appears more frequently in the TERTm group than in the TERTw group, which is potentially ascribed to the abnormal increase in cell membrane metabolism, energy depletion, anaerobic glycolysis, and the cell necrosis caused by the increased cell proliferation and mitosis of the tumor. Though Lip peak was verified to be associated with the activity of tumor necrosis [38], there was no predictive value demonstrated in our research, which may be due to the Lip peak being more sensitive to the placement of voxels, thus affecting the definitive results [34]. Therefore, it is necessary to conduct a further study on the Lip feature for predicting TERT promoter mutations. Age and gender were also taken into consideration for model development. From the results of univariate analyses, the probability of TERT mutation upregulated with every additional year of age (4% and 6% in training and validation cohorts, respectively). However, gender was not statistically significant in this study. You et al. [39] analyzed 887 gliomas for TERT promoter mutations based on histological and genetic backgrounds by DNA sequencing, which demonstrated that the frequency of TERT mutations increased with age. From what has been discussed above, TERTm in adults with HGG were more likely to be older, with visible Lac peaks, the higher Cho/Cr indexes, Radscore, and CNV (Figure 7).

Despite the promising results, some limitations of this study were also necessary to be further investigated. Firstly, functional magnetic resonance imaging series were not taken into account in this study, such as diffusion-weighted imaging, dynamic contrast-enhanced, and intravoxel incoherent motion. We believed that the addition of the advanced imaging features would improve the performance of the established radiomics nomogram which was expected to be tried in the next step. Secondly, it has been indicated in the literature that TERT played different roles in different IDH and ATRX phenotypes [38]. However, only the feasibility and methods to predict TERT promoter mutations were explored in our study, and no above-stratified analysis was performed, which was also the emphasis in our next research. Lastly, more advanced machine learning modeling methods was not used, such as support vector machines and neural networks, because the simplest logistic regression we used has yielded satisfactory results.

## 5. Conclusions

In conclusion, Age, Cho/Cr, Lac, CNV, and Radscore were important indicators for TERT promoter mutation predictions in HGG. Furthermore, tumor necrosis seems to be closely related to TERT promoter mutations. Radiomics nomogram based on multiparameter MRI and CNV could effectively predict the TERT promoter mutations in adults who suffered from HGG with high accuracy.

## Figures and Tables

**Table 1:** Characteristics of patients in the training and validation cohorts.

	Training cohort			Validation cohort			*p* value
	TERT_w_	TERT_m_	*p* value	TERT_w_	TERT_m_	*p* value	
*N*	51	37		22	16		0.995
Age (year)†	53 (18-78)	58 (30-81)	0.033	52 (26-78)	60 (42-80)	0.031	0.986
Gender			0.571			0.120	0.975
Female	19 (37.25%)	16 (43.24%)		11 (50.00%)	4 (25.00%)		
Male	32 (62.75%)	21 (56.76%)		11 (50.00%)	12 (75.00%)		
Grade			0.170			0.366	0.327
III	21 (41.18%)	10 (27.03%)		7 (31.82%)	3 (18.75%)		
IV	30 (58.82%)	27 (72.97%)		15 (68.18%)	13 (81.25%)		
Side			0.384			0.050	0.219
Left	27 (52.94%)	15 (40.54%)		17 (77.27%)	7 (43.75%)		
Right	20 (39.22%)	20 (54.05%)		3 (13.64%)	8 (50.00%)		
Both	4 (7.84%)	2 (5.41%)		2 (9.09%)	1 (6.25%)		
Location			0.048			0.028	0.421
Frontal	23 (45.10%)	7 (18.92%)		8 (36.36%)	1 (6.25%)		
Temporal	16 (31.37%)	23 (62.16%)		4 (18.18%)	11 (68.75%)		
Parietal	7 (13.73%)	3 (8.11%)		4 (18.18%)	2 (12.50%)		
Occipital	2 (3.92%)	2 (5.41%)		2 (9.09%)	1 (6.25%)		
Other	3 (5.88%)	2 (5.41%)		4 (18.18%)	1 (6.25%)		
NeV†	0.42 (0.00-2.59)	0.86 (0.04-4.44)	0.243	0.41 (0.00-2.61)	0.95 (0.08-4.05)	0.102	0.559
EnV^†^	2.62 (0.02-41.70)	1.93 (0.03-37.17)	0.715	3.02 (0.20-28.36)	1.97 (0.11-9.99)	0.115	0.633
EdV^†^	1.31 (0.01-56.46)	2.88 (0.03-37.17)	0.162	1.16 (0.04-65.77)	2.15 (0.45-17.06)	0.856	0.827
CNV (%)†	14.71 (0.00-40.44)	43.48 (14.91-84.49)	<0.001	14.38 (0.00-38.38)	37.64 (11.54-71.20)	<0.001	0.264
ONV (%)†	12.44 (0.00-39.55)	15.42 (4.55-29.16)	0.196	6.75 (0.00-32.33)	17.98 (1.76-28.18)	0.091	0.197
Cho/NAA	4.65 (3.14)	4.40 (1.89)	0.670	4.22 (1.51)	4.80 (2.79)	0.413	0.868
Cho/Cr	2.75 (1.06)	3.39 (1.53)	0.023	2.54 (0.68)	3.19 (1.04)	0.025	0.390
Lip			0.616			0.152	0.944
No	33 (64.71%)	22 (59.46%)		16 (72.73%)	8 (50.00%)		
Yes	18 (35.29%)	15 (40.54%)		6 (27.27%)	8 (50.00%)		
Lac			0.025			0.034	0.370
No	33 (64.71%)	15 (40.54%)		17 (77.27%)	7 (43.75%)		
Yes	18 (35.29%)	22 (59.46%)		5 (22.73%)	9 (56.25%)		

†Data were the median(min-max); the remainder were mean (standard deviation) or number (%). *p* value <0.05 was considered a significant difference. NeV: necrotic volume of tumor; EnV: volume of enhanced portion of tumor; EdV: volume of edema around tumor; CNV: necrotic volume percentage of core; ONV: necrotic volume percentage of overall; Cho/NAA: the ratio of N-acetylaspartic acid to creatine in MRS; Cho/Cr: the ratio of choline to creatine in MRS; Lip: lipid peak of MRS; Lac: lactate peak of MRS; TERTm: the TERT promoter mutations; TERTw: the wild type of TERT promoter.

**Table 2:** Results of dimensionality reduction and the ICC for each feature.

Abbreviation	VOI	Image type	Feature class	Feature name	ICC	
					Intra-	Inter-
t1c_necrosis_glszm_6	Necrosis	T1c_original	glszm	GrayLevelNonUniformity	0.977	0.951
t1c_necrosis_wavelet_448	Necrosis	T1c_wavelet_HLH	glszm	SizeZoneNonUniformityNormalized	0.887	0.856
t2f_necrosis_wavelet_169	Necrosis	T2f_wavelet_LHL	glszm	SizeZoneNonUniformityNormalized	0.908	0.889
necrosis_shap_6	Necrosis	Original	Shape	LeastAxisLength	0.922	0.901
t1c_enhanced_wavelet_217	Enhanced	T1c_wavelet_LHH	glcm	MCC	0.949	0.938
t2f_necrosis_wavelet_660	Necrosis	T2f_wavelet_LLL	gldm	LargeDependenceEmphasis	0.979	0.952

T1c: contrast-enhanced T1-weighted; T2f: T2-weighted flair; VOI: the volume of interest; ICC: intraclass correlation coefficient; Intra-: intra-observer agreement; Inter-: inter-observer agreement.

**Table 3:** Univariate and multivariate analyses of TERT promoter mutations.

	Univariate		Multivariate	
	OR (95% CI)	*p* value	OR (95% CI)	*p* value
Age	1.04 (1.00, 1.08)	0.038	1.06 (1.01, 1.12)	0.025
Gender	0.78 (0.33, 1.85)	0.571		
Grade	1.89 (0.76, 4.72)	0.173		
Side	2.00 (0.33, 12.18)	0.452		
Location	2.19 (0.30, 15.85)	0.437		
NeV	1.38 (0.80, 2.35)	0.245		
EnV	0.99 (0.93, 1.05)	0.712		
EdV	1.04 (0.98, 1.11)	0.197		
CNV (%)	1.15 (1.09, 1.22)	<0.001	1.12 (1.06 1.18)	<0.001
ONV (%)	1.04 (0.98, 1.10)	0.196		
Cho/NAA	0.96 (0.82, 1.14)	0.668		
Cho/Cr	1.48 (1.04, 2.09)	0.028	2.71 (1.15, 3.25)	0.012
Lip	1.25 (0.52, 2.99)	0.616		
Lac	2.69 (1.12, 6.43)	0.026	3.14 (1.01, 9.74)	0.048
Radscore	2.72 (1.78, 4.14)	<0.001	2.04 (1.51, 2.77)	<0.001

OR: odds ratios; CI: confidence intervals; NeV: necrotic volume of tumor; EnV: volume of enhanced portion of tumor; EdV: volume of edema around tumor; CNV: necrotic volume percentage of core; ONV: necrotic volume percentage of overall; Cho/NAA: the ratio of N-acetylaspartic acid to creatine in MRS; Cho/Cr: the ratio of choline to creatine in MRS; Lip: lipid peak of MRS; Lac: lactate peak of MRS; Radscore: radiomics feature. *p* value <0.05 was considered significant difference.

**Table 4:** Performance of 4 models for TERT promoter mutations prediction.

	Training cohort							Validation cohort						
	AUC (95% CI)	SEN	SPE	ACC	PPV	NPV	Cutoff	AUC (95% CI)	SEN	SPE	ACC	PPV	NPV	Cutoff
Model A	0.955 (0.899-0.979)	0.947	0.840	0.886	0.818	0.955	-0.652	0.889 (0.746-0.959)	0.750	0.909	0.842	0.857	0.833	-0.652
Model B	0.917 (0.840-0.959)	0.973	0.745	0.841	0.735	0.974	-1.14	0.868 (0.668-0.923)	0.813	0.818	0.816	0.765	0.857	-1.14
Model C	0.841 (0.802-0.913)	0.919	0.725	0.807	0.708	0.925	-0.798	0.747 (0.586-0.891)	0.750	0.727	0.737	0.667	0.800	-0.798
Model D	0.832 (0.798-0.919)	0.914	0.717	0.795	0.681	0.927	24.899	0.714 (0.608-0.829)	0.867	0.652	0.737	0.619	0.882	24.899

Abbreviations: Model A: Age+Lac+Cho/Cr+Radscore +CNV; Model B: Age+Lac+Cho/Cr+Radscore; Model C: Radscore; Model D: CNV; AUC: area under the curve; SEN: sensitivity; SPE: specificity; ACC: accuracy; PPV: positive predictive value; NPV: negative predictive value; CI: confidence intervals. The bootstrap resampling method was adopted for 95% CI and the significance test of AUC (times = 500). The cutoff value was determined based on the output value of the radiomics nomogram in the training cohort.

## Data Availability

The data that support the findings of this study are available on request from the corresponding author (Guangyao Wu). The data are not publicly available because of the aforementioned data containing information that could compromise research participant privacy.

## Supplementary Materials

Supplementary Materials Supplementary S1: The acquisition parameters of MRI. Supplementary S2: Procedures and parameters for tumor separation. Supplementary S3: Radiomics feature extraction. Supplementary S4: Statistical analysis. Supplementary S5: Radscore formula. Supplementary S6: Performance of the models for predicting TERT promoter mutations.
