# Supplementary material for: Noninvasive Prediction of TERT Promoter Mutations in High-Grade Glioma by Radiomics Analysis Based on Multiparameter MRI
Source: Biomed Res Int. 2020 May 15;2020:3872314. doi: 10.1155/2020/3872314 (PMC7245686; doi:10.1155/2020/3872314)
Supplement: Supplementary Materials — Supplementary S1: The acquisition parameters of MRI. Supplementary S2: Procedures and parameters for tumor separation. Supplementary S3: Radiomics feature extraction. Supplementary S4: Statistical analysis. Supplementary S5: Radscore formula. Supplementary S6: Performance of the models for predicting TERT promoter mutations. [file 3872314.f1.pdf]

Supplementary S1: The acquisition parameters of MRI

All preoperative MRI was performed on 3.0T MR scanners (Magneto Trio, Siemens, Germany) with an eight-channel head coil. Imaging protocols included contrast-enhanced T1-weighted (CE-T1w), T2-weighted imaging fluid-attenuated inversion recovery (T2flair), T1-weighted (T1w), T2-weighted (T2w), and Magnetic Resonance Spectroscopy (MRS). After weight-adjusted injection of a gadolinium-based contrast agent at a dose of 0.1 mmol per kilogram, CE-T1w were performed. MRS was located in the largest solid level via T2WI or CE-T1w, including the solid tumor and peripheral edema areas.

**Sequences**	**Orientation**	**TE (ms)**	**TR (ms)**	**TI (ms)**	**Slice thickness (mm)**	**Intersection gap (mm)**	**FOV (cm × cm)**	**Matrix**
T1WI	Axial	4-8	150-300	-	3.0	3.0	24.0 × 24.0	384 × 288
T2WI	Axial	80-110	3200-5000	-	3.0	3.0	24.0 × 24.0	384 × 288
T2-FLAIR	Axial	85-110	6000-8000	2000	3.0	3.0	24.0 × 24.1	384 × 288
CE-T1w	3D	3-5	1900-2200	-	1.0	1.0	25.6 × 24.0	256 × 256
MRS	2D	135	1700	NEX=1		Voxel=10 × 10 × 10 mm^3		

Abbreviations: TE, echo time; TR, repetition time; TI, inversion time; FOV, field of vision.

Supplementary S2: Procedures and parameters for tumor separation

Three heterogeneous regions were plotted by semi-automatic segmentation based on the "Grow from seed" and "Threshold" functions in the Segment Editor module. The tumor boundaries were determined in CE-T1w and T2w. For CE-T1w, 3 different types were segmented: the enhanced lesion inclusive of necrosis (Tumor Core), enhanced and necrosis. T1W and T2w were adopted to assist CE-T1w in adjusting the above areas, especially in the non-enhanced tumor section. Peritumoral edema regions were outlined on the T2flair and T2w images. To avoid the overlap of necrosis, enhanced and edema, " Logical operators " module subtraction operation were adopted.

In the "threshold" module:

The threshold range of enhancement area was set as 200-1000, and the threshold range of necrosis area was set as 0-200 in CE-T1w series. In the T2flair images, the threshold range of edema area was set as: 400-1000.

In the "Grow from seed" module:

Three types of seed points were set on the necrosis, enhancement and edema zones respectively. The placement of seed points in the axial, sagittal, and coronal positions can accelerate the accuracy and efficiency of tumor segmentation. Seed points should be set in axial position, sagittal position and coronal position to accelerate the accuracy and efficiency of tumor segmentation. The number of seed points could be selected flexibly according to the size of the tumor. Since there were many layers of the tumor, more seed points could be set.

Supplementary S3: Radiomics Feature extraction

A total of 851 radiomics features were used, mainly derived from pyradiomics. For detailed description of features, please refer to: https://pyradiomics.readthedocs.io/en/latest/features.html#radiomics-features-label. They are subdivided into the following classes:

- Shape-based (14 features)
- First Order Statistics (18 features)
- Texture feature (75 features)
  - Gray Level Dependence Matrix (14 features)
  - Gray Level Cooccurence Matrix (24 features)
  - Gray Level Run Length Matrix (16 features)
  - Gray Level Size Zone Matrix (16 features)
  - Neighbouring Gray Tone Difference Matrix (5 features)
- Wavelet feature (93* 8= 744 features)
  Wavelet, the value in brackets indicates the filters (H: High pass filter, L: Low pass filter) applied in the x, y and z directions, respectively.
  - LLL
  - LLH
  - LHL
  - LHH
  - HLL
  - HLH
  - HHL
  - HH

■ Shape-based Features

1. Voxel Volume
2. Maximum3Ddiameter
3. MeshVolume
4. MajorAxisLength
5. Sphericity
6. LeastAxisLength
7. Elongation
8. SurfaceVolumeRatio
9. Maximum2DDiameterSlice
10. Flatness
11. SurfaceArea
12. SurfaceArea
13. Maximum2DDiameterColumn
14. Maximum2DDiameterRow

■ First Order Statistics

1. InterquartileRange
2. Skewness
3. Uniformity
4. Median
5. Energy
6. RobustMeanAbsoluteDeviation
7. MeanAbsoluteDeviation
8. TotalEnergy
9. Maximum
10. RootMeanSquared
11. 90Percentile
12. Minimum
13. Entropy
14. Range
15. Variance
16. 10Percentile
17. Kurtosis
18. Mean

■ Gray Level Dependence Matrix (GLDM)

1. GrayLevelVariance
2. HighGrayLevelEmphasis
3. DependenceEntropy

4. DependenceNonUniformity
5. GrayLevelNonUniformity
6. SmallDependenceEmphasis
7. SmallDependenceHighGrayLevelEmphasis
8. DependenceNonUniformityNormalized
9. LargeDependenceEmphasis
10. LargeDependenceLowGrayLevelEmphasis
11. DependenceVariance
12. LargeDependenceHighGrayLevelEmphasis
13. SmallDependenceLowGrayLevelEmphasis
14. LowGrayLevelEmphasis

■ Gray Level Co-occurrence Matrix (GLCM)

1. JointAverage
2. SumAverage
3. JointEntropy
4. ClusterShade
5. MaximumProbability
6. Idmn
7. JointEnergy
8. Contrast
9. DifferenceEntropy
10. InverseVariance
11. DifferenceVariance
12. Idn
13. Idm
14. Correlation
15. Autocorrelation
16. SumEntropy
17. MCC
18. SumSquares
19. ClusterProminence
20. Imc2
21. Imc1
22. DifferenceAverage
23. Id
24. ClusterTendency

■ Gray Level Run Length Matrix (GLRLM)

1. ShortRunLowGrayLevelEmphasis
2. GrayLevelVariance
3. LowGrayLevelRunEmphasis
4. GrayLevelNonUniformityNormalized
5. RunVariance
6. GrayLevelNonUniformity
7. LongRunEmphasis
8. ShortRunHighGrayLevelEmphasis
9. RunLengthNonUniformity
10. ShortRunEmphasis
11. LongRunHighGrayLevelEmphasis
12. RunPercentage
13. LongRunLowGrayLevelEmphasis
14. RunEntropy
15. HighGrayLevelRunEmphasis
16. RunLengthNonUniformityNormalized

■ Gray Level Size Zone Matrix (GLSZM)

1. GrayLevelVariance
2. ZoneVariance
3. GrayLevelNonUniformityNormalized
4. SizeZoneNonUniformityNormalized
5. SizeZoneNonUniformity
6. GrayLevelNonUniformity
7. LargeAreaEmphasis
8. SmallAreaHighGrayLevelEmphasis
9. ZonePercentage
10. LargeAreaLowGrayLevelEmphasis
11. LargeAreaHighGrayLevelEmphasis
12. HighGrayLevelZoneEmphasis
13. SmallAreaEmphasis
14. LowGrayLevelZoneEmphasis
15. ZoneEntropy
16. SmallAreaLowGrayLevelEmphasis

■ Neighbouring Gray Tone Difference Matrix (NGTDM)

1. Coarseness
2. Complexity
3. Strength
4. Contrast
5. Busyness

Supplementary S4: Statistical analysis

In our study, continuous variables (age, radscore, etc.) were assessed using Student's t-test or Mann-Whitney U test to determine whether there were differences between groups, while the categorical variables (gender, location, etc.) were analyzed by chi-square or Fisher test. The following R packages were used: the “glmnet” package was used for the LASSO-logistic regression, the “psych” package was used for the ICC test, the “rms” and “ggplot2” package was used for the nomograms and calibration curves, the “pROC” package was used for the ROC curves, the “PredictABEL” package was used for NRI and IDI, and the “rmda” package was used to implement the DCA. A two-tailed $p < 0.05$ was considered to be statistically significant in all analyses.

Supplementary S5: Radscore formula

A radscore formula was established according to the coefficients of the six features obtained previously, which had a high predictive performance on the TERT promoter mutation. As followed:

$$\textbf{Radscore} = -2.6095 + 0.0973 \times \text{necrosis\_shap\_6} + 0.4058 \times \text{t1c\_necrosis\_wavelet\_448} - 0.0705 \times \text{t1c\_necrosis\_glszm\_6} + 0.8273 \times \text{t1c\_enhanced\_wavelet\_217} + 0.4634 \times \text{t2f\_necrosis\_wavelet\_169} + 0.9501 \times \text{t2f\_necrosis\_wavelet\_660}$$

Performance of 6 radiomics features for TERT promoter mutations prediction.

	**Training cohort**							**Validation cohort**						
	AUC (95%CI)	SEN	SPE	ACC	PPV	NPV	Cutoff	AUC (95%CI)	SEN	SPE	ACC	PPV	NPV	Cutoff
**t1c_necrosis_glszm_6**	0.766 (0.670- 0.865)	0.784	0.765	0.773	0.707	0.829	32.651	0.659 (0.485-0.823)	0.875	0.546	0.684	0.583	0.857	32.651
**t1c_necrosis_wavelet_448**	0.752 (0.650-0.832)	0.784	0.648	0.705	0.617	0.805	0.732	0.661 (0.471-0.823)	0.438	0.909	0.711	0.778	0.690	0.732
**t2f_necrosis_wavelet_169**	0.750 (0.634-0.834)	0.811	0.628	0.705	0.612	0.821	0.689	0.690 (0.505-0.840)	0.688	0.682	0.684	0.611	0.750	0.689
**necrosis_shap_6**	0.774(0.673-0.858)	0.628	0.919	0.750	0.642	0.914	17.572	0.698 (0.530-0.837)	0.938	0.455	0.658	0.556	0.909	17.572
**t1c_enhanced_wavelet_217**	0.686(0.570-0.799)	0.784	0.628	0.693	0.604	0.800	0.198	0.640 (0.447-0.792)	0.438	0.909	0.711	0.778	0.690	0.198
**t2f_necrosis_wavelet_660**	0.773 (0.675-0.874)	0.622	0.941	0.807	0.885	0.774	126.741	0.677 (0.514-0.839)	0.813	0.591	0.684	0.591	0.813	126.741

Abbreviations: AUC, area under the curve; SEN, sensitivity; SPE, specificity; ACC, accuracy; PPV, positive predictive value; NPV, negative predictive value; CI, confidence intervals. The bootstrap resampling method was adopted for 95%CI and the significance test of AUC (times= 500). The cutoff value was determined based on the output value of the radiomics nomogram in the training cohort.

Supplementary S6:
Performance of the models for predicting TERT promoter mutations

	**Training cohort**							**Validation cohort**						
**MODEL**	**AUC (95%CI)**	**SEN**	**SPE**	**ACC**	**PPV**	**NPV**	**Cutoff**	**AUC (95%CI)**	**SEN**	**SPE**	**ACC**	**PPV**	**NPV**	**Cutoff**
**AGE**	0.613 (0.508-0.742)	0.838	0.471	0.625	0.534	0.800	51.500	0.605 (0.499-0.823)	0.824	0.524	0.658	0.583	0.786	51.500
**MRS**	0.660 (0.551-0.788)	0.784	0.549	0.648	0.558	0.778	-0.621	0.767 (0.585-0.889)	0.500	0.727	0.632	0.571	0.667	-0.621
**RADSCORE**	0.841 (0.802-0.913)	0.919	0.725	0.807	0.708	0.925	-0.798	0.747 (0.586-0.891)	0.750	0.727	0.737	0.667	0.800	-0.798
**CNV**	0.832 (0.798-0.919)	0.914	0.717	0.795	0.681	0.927	24.899	0.714 (0.608-0.829)	0.867	0.652	0.737	0.619	0.882	24.899
**MR**	0.837 (0.782-0.895)	0.974	0.700	0.818	0.712	0.972	-1.029	0.787 (0.630-0.910)	0.750	0.773	0.763	0.706	0.810	-1.029
**MC**	0.851 (0.808-0.927)	0.784	0.902	0.852	0.853	0.852	0.302	0.779 (0.659-0.848)	0.842	0.579	0.711	0.667	0.786	0.302
**MA**	0.757 (0.681-0.811)	0.622	0.863	0.761	0.767	0.759	-0.015	0.794 (0.675-0.838)	0.647	0.810	0.737	0.733	0.739	-0.015
**AR**	0.870 (0.819-0.943)	0.946	0.765	0.841	0.745	0.951	-0.444	0.762 (0.589-0.891)	0.750	0.773	0.763	0.706	0.810	-0.444
**AC**	0.913 (0.860-0.966)	0.865	0.843	0.852	0.800	0.896	-0.274	0.818 (0.728-0.877)	0.765	0.810	0.789	0.765	0.810	-0.274
**RC**	0.901 (0.867-0.967)	0.921	0.820	0.864	0.795	0.932	-0.489	0.832 (0.690-0.927)	0.875	0.727	0.789	0.700	0.889	-0.489
**MAR**	0.917 (0.840-0.959)	0.973	0.745	0.841	0.735	0.974	-1.14	0.868 (0.668-0.923)	0.813	0.818	0.816	0.765	0.857	-1.14
**MAC**	0.911 (0.840-0.953)	0.811	0.902	0.864	0.857	0.868	0.445	0.811 (0.735-0.921)	0.688	0.864	0.789	0.786	0.792	0.445
**MRC**	0.905 (0.875-0.957)	0.917	0.788	0.841	0.750	0.932	-1.186	0.856 (0.719-0.916)	0.938	0.682	0.789	0.682	0.938	-1.186
**ARC**	0.913 (0.887-0.963)	0.946	0.765	0.841	0.745	0.951	-0.351	0.834 (0.688-0.939)	0.875	0.773	0.816	0.737	0.895	-0.351
**ARCM**	0.955(0.899- 0.979)	0.947	0.840	0.886	0.818	0.955	-0.652	0.889 (0.746-0.959)	0.750	0.909	0.842	0.857	0.833	-0.652

Model MRS consisted of Lac and Cho/Cr features; CNV: the necrosis volume percentage of core; MR: MRS+RADSCORE; MC:MRS+CNV; MA:MRS+AGE; AR:AGE+RADSCORE; AC:AGE+CNV; RC:RADSCORE+CNV; MAR:MRS+AGE+RADSCORE; MAC:MRS+AGE+CNV; MRC:MRS+RADSCORE+CNV; ARC:AGE+RADSCORE+CNV; ARCM: AGE+RADSCORE+CNV+MRS; AUC: area under the curve; SEN: sensitivity; SPE: specificity; ACC: accuracy; PPV: positive predictive value; NPV: negative predictive value. Bootstrap resampling method was adopted for 95%CI and significance test of AUC (times= 500). The cutoff value was determined based on the output value of radiomics nomogram in the training cohort.
